# NF106: A Neurofibromatosis Clinical Trials Consortium Phase II Trial of the MEK Inhibitor Mirdametinib (PD-0325901) in Adolescents and Adults With NF1-Related Plexiform Neurofibromas

**DOI:** 10.1200/JCO.20.02220

**Published:** 2021-01-28

**Authors:** Brian D. Weiss, Pamela L. Wolters, Scott R. Plotkin, Brigitte C. Widemann, James H. Tonsgard, Jaishri Blakeley, Jeffrey C. Allen, Elizabeth Schorry, Bruce Korf, Nathan J. Robison, Stewart Goldman, Alexander A. Vinks, Chie Emoto, Tsuyoshi Fukuda, Coretta T. Robinson, Gary Cutter, Lloyd Edwards, Eva Dombi, Nancy Ratner, Roger Packer, Michael J. Fisher

**Affiliations:** ^1^ Cincinnati Children's Hospital, Cincinnati, OH; ^2^ NCI, Center for Cancer Research, Bethesda, MD; ^3^ Massachusetts General Hospital, Boston, MA; ^4^ University of Chicago, Chicago, IL; ^5^ Johns Hopkins Medical Center, Baltimore, MD; ^6^ New York University, New York, NY; ^7^ University of Alabama-Birmingham, Birmingham, AL; ^8^ Children's Hospital Los Angeles, Los Angeles, CA; ^9^ Lurie Children's Hospital of Chicago, Chicago, IL; ^10^ Children's National Hospital, Washington, DC; ^11^ Children's Hospital of Philadelphia, Philadelphia, PA

## Abstract

Patients with neurofibromatosis type 1 (NF1) frequently develop plexiform neurofibromas (PNs), which can cause significant morbidity. We performed a phase II trial of the MAPK/ERK kinase inhibitor, mirdametinib (PD-0325901), in patients with NF1 and inoperable PNs. The primary objective was response rate based on volumetric magnetic resonance imaging analysis.

**METHODS:**

Inclusion criteria included age ≥ 16 years and a PN that was either progressive or causing significant morbidity. First-dose pharmacokinetics were performed. Patients completed patient-reported outcome measures. Patients received mirdametinib by mouth twice a day at 2 mg/m^2^/dose (maximum dose = 4 mg twice a day) in a 3-week on/1-week off sequence. Each course was 4 weeks in duration. Evaluations were performed after four courses for the first year and then after every six courses. Patients could receive a maximum of 24 total courses.

**RESULTS:**

Nineteen patients were enrolled, and all 19 received mirdametinib. The median age was 24 years (range, 16-39 years); the median baseline tumor volume was 363.8 mL (range, 3.9-5,161 mL). Eight of the 19 patients (42%) achieved a partial response of the target PN by course 12, and 10 (53%) had stable disease. One patient (5%) developed progressive disease at course 8. Significant and durable decreases were observed in pain ratings.

**CONCLUSION:**

To our knowledge, this analysis represents the first characterization of the activity and pharmacokinetics of mirdametinib in patients with NF1 and PNs and is the first published response study for MAPK/ERK kinase inhibitors in adults with NF1 and PNs. Mirdametinib given at 2 mg/m^2^/dose (maximum dose, 4 mg) twice daily in a 3-week on/1-week off sequence resulted in a 42% partial response rate with preliminary evidence of reduction in pain.

## INTRODUCTION

Neurofibromatosis type 1 (NF1) is a common autosomal dominant disorder with an incidence of 1:2,700^1^ caused by a germline pathogenic variant in the *NF1* tumor suppressor gene. NF1 is characterized by progressive cutaneous, neurological, skeletal, and neoplastic manifestations; patients have a 40% risk of developing plexiform neurofibromas (PNs). PNs can cause significant disfigurement, compression of vital structures, neurologic dysfunction, and pain, which can negatively affect quality of life (QOL).^2^ Until recently, the only management strategy was surgical resection, which is often difficult because of the infiltrative nature of the tumor. New evidence suggests that inhibition of the MAPK/ERK kinase (MEK) pathway can lead to significant PN shrinkage^3^ and clinical benefit^4^ in children with NF1.

**CONTEXT**

**Key Objective**
Will the MAPK/ERK kinase inhibitor mirdametinib (PD-0325901) shrink neurofibromatosis type 1 (NF1)–related plexiform neurofibromas (PNs) in adolescents and adults?
**Knowledge Generated**
Treatment with mirdametinib in adolescent and adult patients with NF1 and symptomatic or growing, inoperable PNs in a 3-week on/1-week off sequence was demonstrated to be active and safe. Mirdametinib demonstrated a 42% partial response rate, defined as at least 20% tumor shrinkage by volume as compared with baseline.
**Relevance**
Mirdametinib can be an effective treatment for adolescents and adults with symptomatic or growing, inoperable NF1-related PNs.

The mitogen-activated protein kinase (MAPK) pathway regulates multiple critical cellular functions including growth and senescence^5^; dysregulation of this pathway leads to activation of both extracellular signal–regulated protein kinase (ERK) and MEK. The *NF1* gene encodes neurofibromin, and neurofibromin loss in tumor cells leads to dysregulated Ras signaling with hyperactivation of downstream Ras effectors, including MEK.

Treatment with mirdametinib (PD-0325901), a highly specific noncompetitive MEK inhibitor (MEKi), resulted in shrinkage of PN in a majority of *Nf1* genetically engineered mice,^6^ even at low doses. Thus, we evaluated mirdametinib in a phase II clinical trial for patients with NF1 and symptomatic or growing, inoperable PNs.

## METHODS

### Study Design and Population

Patients were enrolled at NF Clinical Trials Consortium sites. Inclusion criteria included age ≥ 16 years with NF1 and an unresectable PN either with significant progression in the past year (defined as ≥ 20% increase in the volume, ≥ 13% increase in the product of the two longest perpendicular diameters, or ≥ 6% increase in the longest diameter) or with PN-related significant morbidity (Table 1). PNs were at least 3 mL and amenable to volumetric magnetic resonance imaging (MRI) analysis; central review was performed in real time. Exclusion criteria included prior therapy with a MEKi. Other eligibility and exclusion criteria are given in Appendix Table A1 (online only). Patients who had received prior therapy required an adequate washout period. The primary objective was to evaluate the response rate to mirdametinib based on volumetric MRI analysis.

**TABLE 1.**
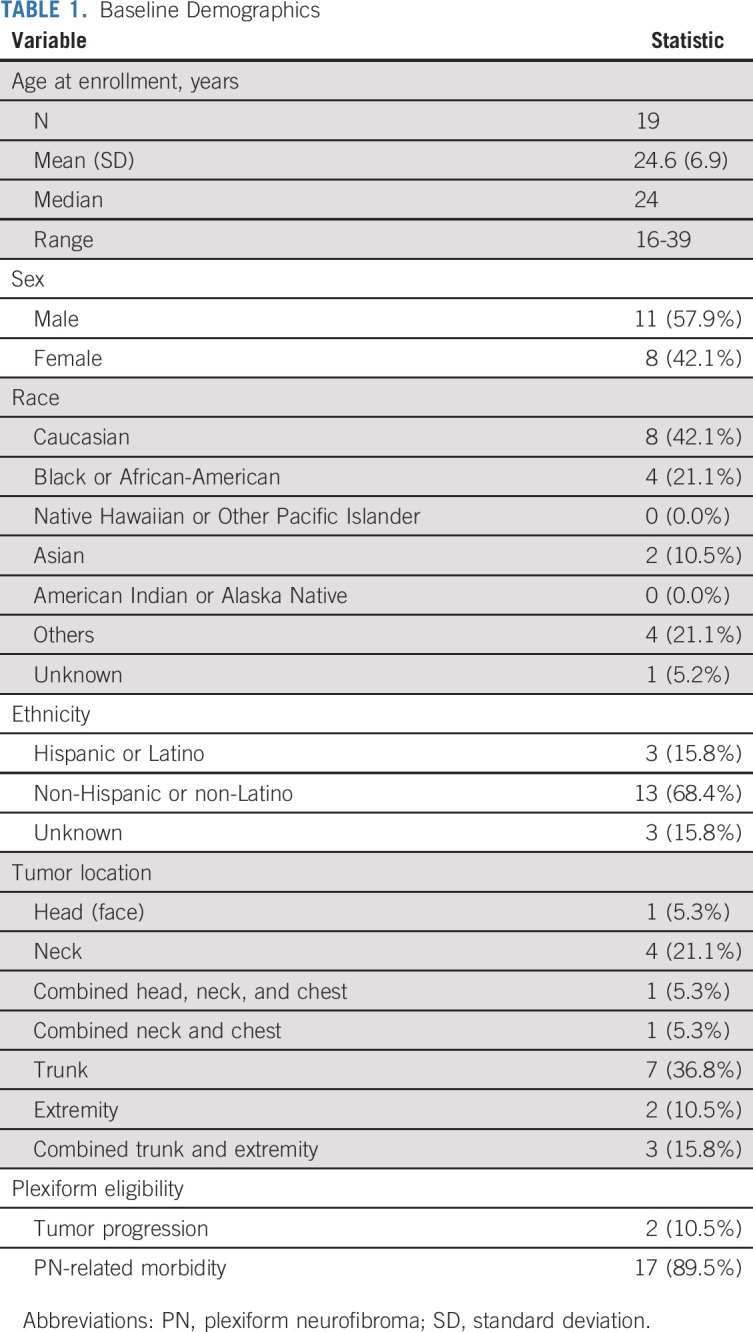
Baseline Demographics

### Therapy

Patients received mirdametinib orally twice a day (BID) at 2 mg/m^2^/dose (maximum dose of 4 mg BID; capsules swallowed whole) in a 3-week on/1-week off sequence because of concerns of musculoskeletal, neurologic, and ocular toxicity seen at doses > 10 mg BID for adults with malignancy^7,8^ and based on evidence of PN shrinkage even with low doses.^6^ Patients could receive a maximum of 24 four-week courses. Patients were removed from therapy for mirdametinib-related dose limiting toxicity (DLT), progressive disease (PD), or lack of partial response (PR; defined as a ≥ 20% reduction in tumor volume compared with baseline) by the end of 12 courses. Patients without at least 15% reduction in target tumor volume by the end of course 8 were removed from protocol therapy for safety concerns, as it was believed that the likelihood of achieving a response by 12 courses was minimal.

DLTs (hematologic or nonhematologic) were defined as either ≥ grade 3 or clinically significant grade 2 toxicities that did not resolve within 72 hours of maximal medical management. Patients who experienced a DLT that was possibly, probably, or definitely related to mirdametinib held drug until the toxicity was completely resolved. If toxicity resolved within 2 weeks, mirdametinib was restarted at a lower dose (Appendix Table A2, online only). Toxicities requiring removal from protocol therapy included: any mirdametinib-related DLT that recurred on a reduced dose (ie, no patient could be dose-reduced more than once); any ≥ grade 3 mirdametinib–related toxicity that did not resolve to ≤ grade 1 within 2 weeks; development of retinal vein occlusion; and development of glaucoma, intraocular pressure > 21 mm Hg, or any significant abnormality on ophthalmic examination.

### Study Evaluations

Patients underwent the following study evaluations at enrollment and after courses 4, 8, and 12, and then after courses 18 and 24 for those who continued therapy.

#### Assessment of PN volume.

Patients underwent noncontrast axial and coronal short-TI inversion recovery MRI at the designated time points. Response was evaluated centrally at the National Cancer Institute based on changes in tumor volume, as previously reported.^9^ PD was defined as a ≥ 20% increase in volume compared with baseline and PR as ≥ 20% reduction in the volume of the target PN.

#### Safety monitoring.

Safety monitoring included physical examination and laboratory studies (including blood counts, comprehensive metabolic panel, and creatine phosphokinase). Patients had an ophthalmology evaluation after courses 1 and 2, then every two courses for the first year, and every three courses in year 2. Adverse events (AEs) were graded according to National Cancer Institute Common Terminology Criteria for Adverse Events v4.0. Participants were considered evaluable for toxicity if they received at least one dose of study drug and were removed from treatment for toxicity or completed one full course of therapy. A priori, we stated that mirdametinib was worthy of further study in this population if ≥ 25% of participants achieved PR after 12 courses without clinically significant toxicity.

#### Patient-reported outcome measures.

Patients completed the following patient-reported outcome (PRO) measures at the above time points.

The Numerical Rating Scale-11^10^ is a self-report measure of pain intensity that was adapted to assess PN-related pain for NF1 clinical trials.^11^ Patients were asked to choose their most important tumor pain and rate it from 0 (no pain) to 10 (worst pain) in the past week. The same tumor pain was to be rated at each evaluation.

The Brief Pain Inventory Pain Interference subscale^12^ assesses the impact of pain on daily functioning in seven areas from 0 (does not interfere) to 10 (completely interfered) in the past week; it yields a mean total score.

The Pediatric Quality of Life Inventory NF1 module is a disease-specific health-related quality-of-life (QOL) measure^13^ assessing 16 domains. Responses on a five-point Likert scale are transformed to a scale of 0-100 (higher scores = better QOL); it produces mean domain and total scores.

#### Pharmacokinetic analysis.

Whole blood samples were collected before treatment and at 30 minutes and 1, 2, 3, 4, 6, 8, and 10 hours after the first dose. Samples were assayed for mirdametinib and the active metabolite, PD-0315209 (only contributes about 3% to total MEK inhibition), by liquid chromatography with tandem mass spectrometry detection (Advion BioSciences, Inc, Ithaca, NY). A noncompartmental analysis was performed to estimate area under the concentration-time curve from time 0 to 12 hours (AUC_0-12h_) after a single dose of mirdametinib using Phoenix WinNonlin (Version 8.1; Certara, Princeton, NJ). A population pharmacokinetics (PK) analysis was also performed using nonlinear mixed effect modeling with NONMEM (version 7.2; ICON, Ellicott City, MD) with Perl speaks NONMEM version 3.6.2 and Pirana version 2.7.1 (Certara). Mirdametinib concentration time data were modeled to generate individual PK estimates during steady-state treatment. The apparent clearance and actual dose treated at time point of response assessment were used to estimate total mirdametinib exposure expressed as AUC_0-12h_ to evaluate the relationship of exposure with tumor shrinkage.

### Statistical Analysis

This study used an optimal Simon 2-stage design, with a null hypothesis response rate of 0.05 and an alternative of 0.25, a power of 80%, and a type I error of 0.05. This called for a first-stage sample size of nine participants with expansion up to 19 participants (if at least one of the first nine had a PR) to achieve at least 17 participants evaluable for response.

The pain and QOL data were summarized with descriptive statistics (means, standard deviation [SD]) via SAS version 9.4 (Cary, NC). Changes over time were evaluated using a mixed model approach via Least Squares Means in the total group (N = 19) and in patients achieving a PR (PR group; n = 8) compared with patients not achieving a PR (no-PR group; n = 11). We fit a final linear mixed model that included time and group as fixed effects (interaction of time and group was not significant and was removed). For random effects, we included only an intercept and assumed exchangeable correlation for the outcomes.

## RESULTS

### Patient Characteristics

Twenty-two patients were screened; 19 were enrolled between July 25, 2014, and September 21, 2015. Two patients (10.5%) enrolled with progressive PN and 17 with a PN causing significant morbidity (Table 1). The median age was 24 years (range, 16-39 years). The median baseline tumor volume was 363.8 mL (range, 3.9-5,161 mL). All 19 patients enrolled were evaluable for toxicity and response.

### Tumor Response

**FIG 1.**
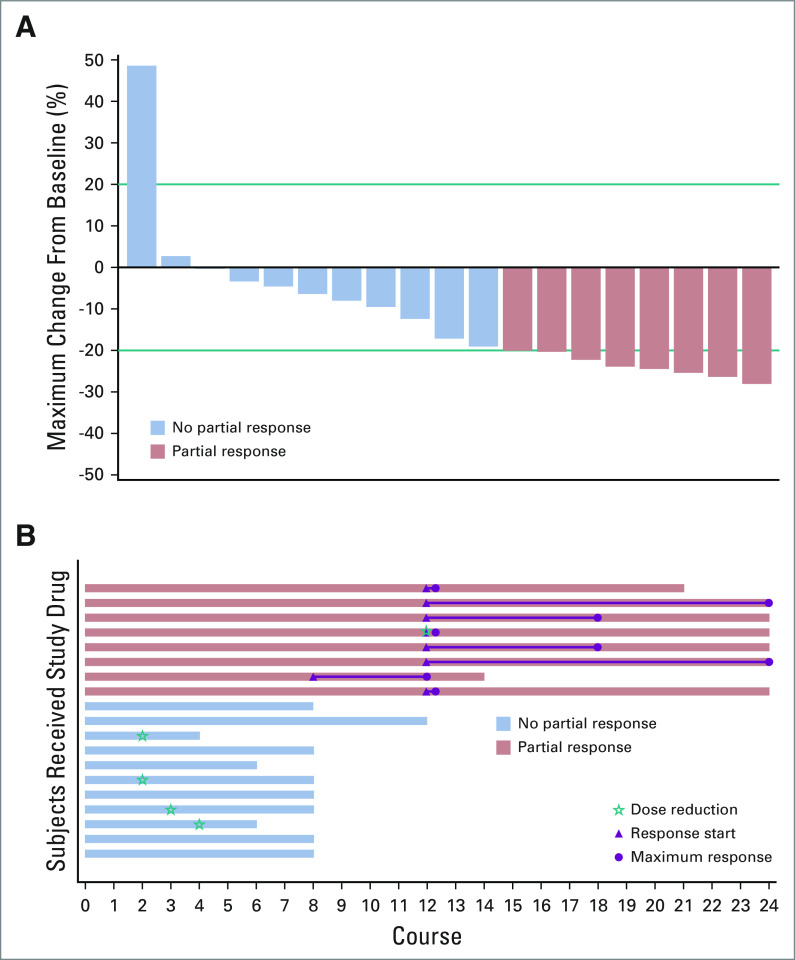
Each patient is represented by a single bar. Blue bars did not achieve a partial response (PR). Red bars achieved a PR. (A) Waterfall plot of maximal tumor volume change by patient. Patients are aligned left to right according to maximal tumor volume change from baseline. There was one patient who had progressive disease, 10 with stable disease, and eight with PR. (B) Swimmers plot of duration of exposure, time to PR, and time to maximum response. Patients are aligned top to bottom from largest response to least response. Length of bar represents duration of exposure. Magenta triangles represent the time a PR was first observed, and magenta circles represent the time of maximum tumor volume change from baseline. Green stars represent time of dose reduction. Note the green star indicating a dose reduction at the same time PR first noted in red bar 4th from the top.

**FIG A1.**
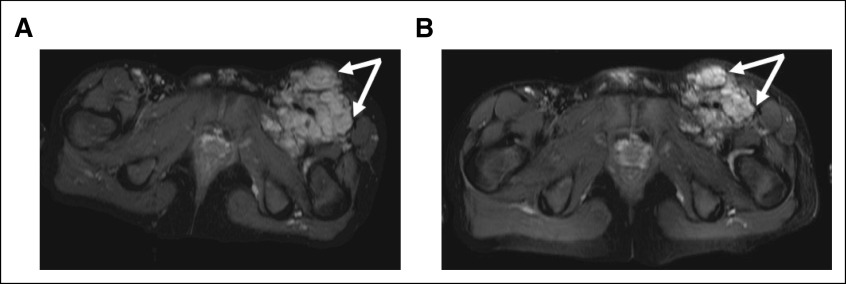
Exemplary response to mirdametinib in a single patient. Axial short-TI inversion recovery magnetic resonance imaging (MRI) sequence shows a plexiform neurofibroma in the left anterior thigh. (A) Example of MRI on enrollment with a volume of 593 mL. (B) Example of MRI after course 12 with a volume of 473 mL.

Eight of the 19 patients (42%) achieved a PR of the target PN by course 12 (Figs 1A and 1B; Appendix Table A3, online only; Appendix Fig A1, online only), and 10 (53%) had stable disease. Thus, we rejected the null hypothesis H0: relative risk ≤ 0.05 in favor of the alternative hypothesis H1: relative risk ≥ 0.25 with *P* < .0001. One patient (5%), who enrolled with a progressive PN, developed PD (tumor growth from 3.9 mL to 5.8 mL) with worsening cervical cord compression at course 8 and stopped protocol therapy. Debulking revealed PN with no evidence of malignant degeneration. Of note, this was one of the two patients enrolled because of PN progression rather than significant morbidity. The median change in tumor volume compared with baseline for all patients was −17.1% (range, −28.0% to +48.7%). Only one patient achieved a PR by the end of course 8; the remaining seven patients achieved PR at course 12 (Fig 1B). One patient had 19% tumor shrinkage at course 8 but elected to stop therapy because of low-grade rash. Maximal tumor response was not achieved until course 18 in two patients and course 24 in two patients (Fig 1B). Of note, only one of the five patients who had a dose reduction achieved a PR, and the dose reduction occurred after the PR was achieved (Fig 1B).

### Patient-Reported Outcomes

All 19 patients completed the PRO measures at baseline, 18 at course 4, 15 at course 8, and nine at course 12. Attrition was due mainly to patients in the no-PR group going off-study (Fig 1B). All eight patients in the PR group completed the measures at each required time point through course 12.

#### Pain intensity.

At baseline, 84% (16 of 19) of patients rated having tumor pain (total sample mean, 4.95; SD, 3.44; range, 0-10) with 69% (11 of 16) reporting moderate to severe levels (ratings ≥ 4). Mean ratings of worst tumor pain intensity decreased significantly in the total sample from baseline to course 4 (*P* = .0075), with both PR and no-PR groups showing a similar trend toward less pain (almost two mean points lower, suggesting clinically meaningful change^14^). In the PR group, decreases in tumor pain intensity remained significantly lower at 12 months (Table 2).

**TABLE 2.**
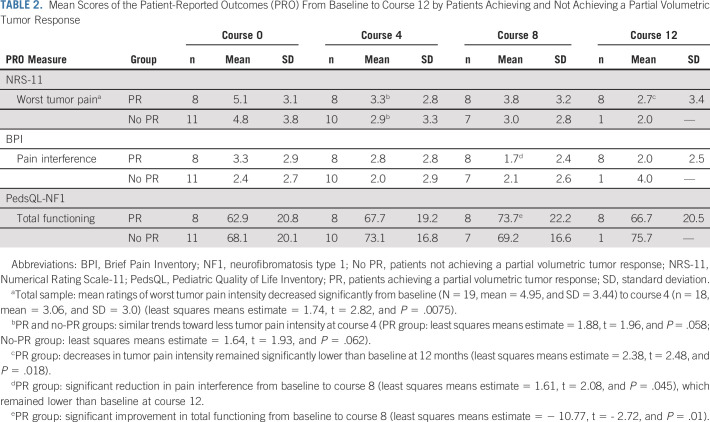
Mean Scores of the Patient-Reported Outcomes (PRO) From Baseline to Course 12 by Patients Achieving and Not Achieving a Partial Volumetric Tumor Response

#### Pain interference.

Mean total Brief Pain Inventory Pain Interference subscale scores were not significantly different from baseline to course 12 at any time point for the total sample. Only the PR group exhibited significant reduction in pain interference from baseline to course 8 (Table 2).

#### Disease-specific QOL.

The total sample showed no significant change in the Pediatric Quality of Life Inventory NF1 total mean score at any evaluation. However, the PR group exhibited significant improvement in total functioning from baseline to course 8 (Table 2).

In the domains (Appendix Table A4, online only), only the PR group rated significant physical changes, including improvements in Movement and Balance at courses 8 and 12 or worsening Skin Irritation at course 4. In the total sample, cognitive functioning mean scores improved significantly at course 8 (*P* = .042).

### Clinical Safety and Tolerability

No patients discontinued treatment because of DLT. One patient developed two treatment-related grade 3 AEs (back and abdominal pain) simultaneously during course 1; the pain resolved upon holding the drug and did not recur at the protocol-mandated reduced dose. There were no grade 4 or 5 AEs. Five patients (26.3%) required dose reductions while on study: for grade 3 abdominal and/or back pain (as described above), grade 1 rash (n = 2), grade 2 nausea (n = 1), and grade 2 fatigue (n = 1). The most common AEs (any grade) were acneiform rash (94.7%), fatigue (57.9%), and nausea (52.6%) (Table 3).

**TABLE 3.**
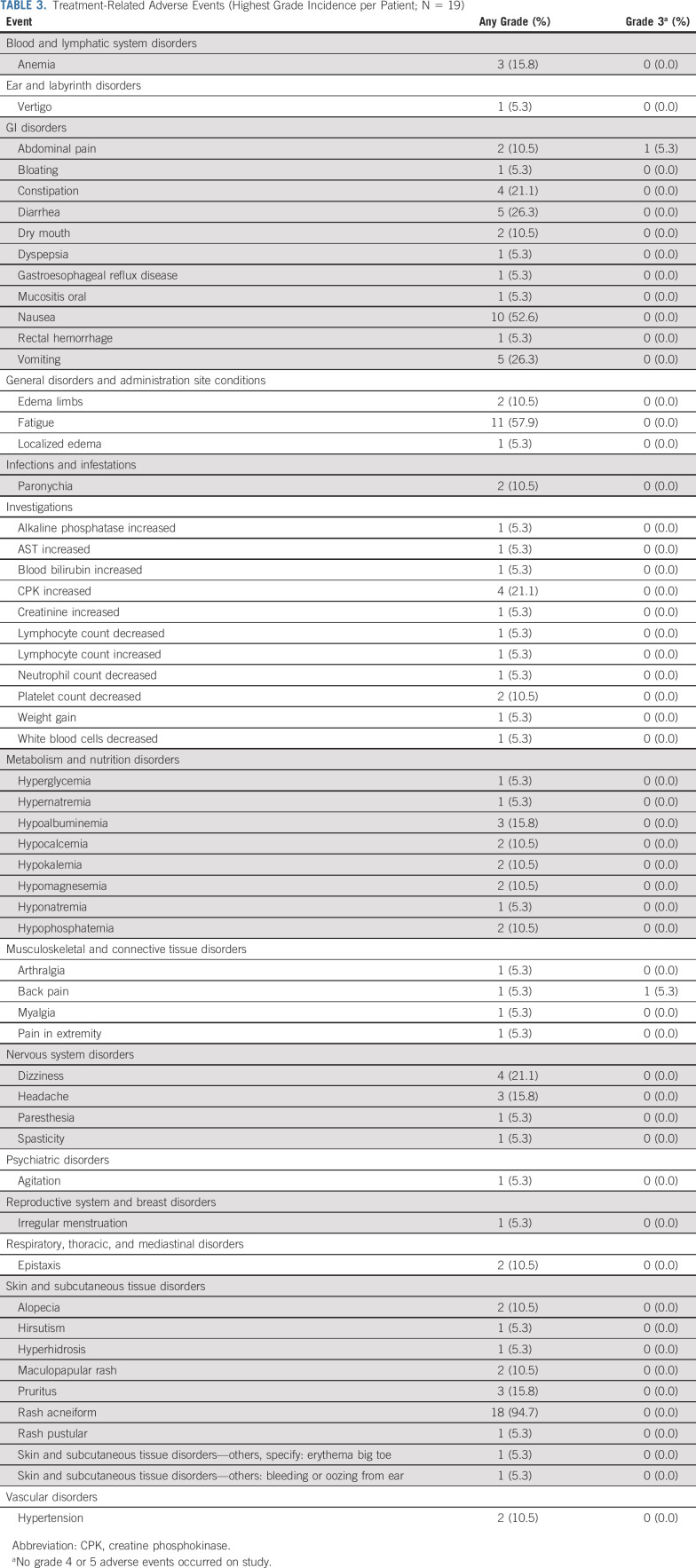
Treatment-Related Adverse Events (Highest Grade Incidence per Patient; N = 19)

Of the 19 participants, six completed all planned therapy, one was removed from study at course 12 because of lack of response, and seven were removed from study at course 8 because of < 15% tumor volume reduction. Five patients withdrew from protocol therapy (two of whom had achieved a PR): four because of low-grade rash perceived to be intolerable and one who felt the study commitments were too challenging.

### PK for Exposure-Response Analysis

The concentration-time profile (PK) data were available for 18 patients around the first dose of course 1. Mean AUC_0-12h_ values (± SD) of mirdametinib and PD-0315209 (metabolite) on day 1 were 443 (± 103) and 184 (± 101) (ng * h/mL), respectively. The mean apparent mirdametinib clearance was 7.6 L/h (± 2.6) and showed a good correlation with both body weight and body surface area (R^2^ of 0.80 and 0.77, respectively).

### Correlation Between Tumor Response and Exposure of Mirdametinib

**FIG 2.**
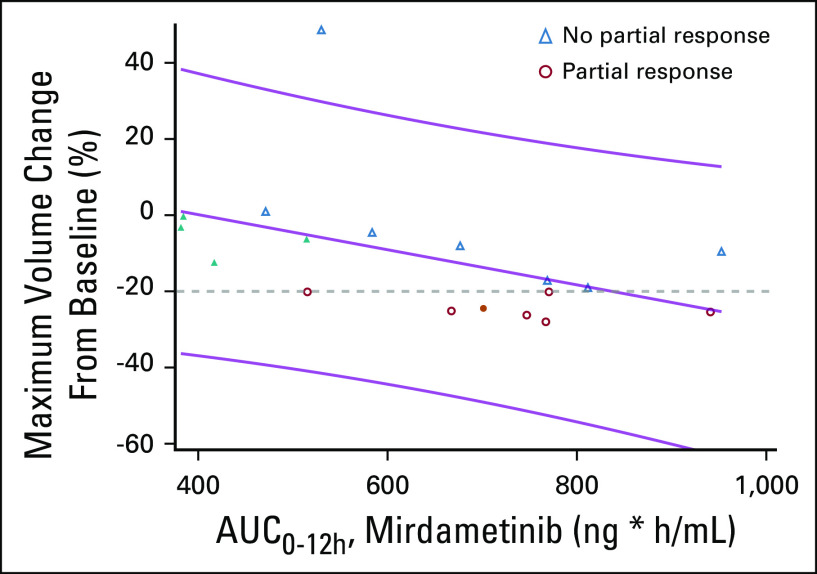
Relationship between the maximum tumor volume change from baseline and the AUC_0-12h_ estimate in steady state (R^2^ = 0.22; *P* = .052). The predicted exposure of mirdametinib at tumor size readout (as measured by AUC_0-12h_) was compared with maximum tumor volume change from baseline in individual patients using linear regression analysis. Blue triangles represent patients who did not achieve a partial response (PR). Red circles are those patients who did achieve a PR; one patient who achieved a PR did not have pharmacokinetics performed. Filled in shapes represent patients who had a dose reduction, only one of whom achieved a PR (although the dose reduction was after the PR was achieved). The patient near the very top of the graph had a small plexiform neurofibroma that significantly progressed in the first eight courses. AUC_0-12h_, area under the concentration-time curve from time 0 to 12 hours.

A time-dependent trend in tumor shrinkage throughout the study was observed. Maximum tumor shrinkage from baseline suggests a positive relationship with mirdametinib exposure (AUC_0-12h_) (R^2^ = 0.22; *P* = .052) (Fig 2). Most responses were seen in patients whose AUC was ≥ 600 (ng * h/mL). PK was available for all five patients who had dose reductions (around their original dose); only one had an AUC ≥ 600 (ng * h/mL), and that patient achieved a PR before the dose reduction.

## DISCUSSION

This analysis represents the first characterization of the activity and PK of mirdametinib and its metabolite in patients with NF1 and PNs. Furthermore, to our knowledge this is the first published response study for MEKi in adults with NF1 and PNs.

We showed that mirdametinib given at 2 mg/m^2^/dose (maximum dose, 4 mg) twice daily in a 3-week on/1-week off sequence results in a 42% PR rate. Although this is a promising response rate, it is lower than the 71%-74% PR rate in the phase I and II trials of the MEKi selumetinib in children with NF1 and inoperable PNs.^3,4^ However, these trials, which had differing eligibility criteria, were not designed to compare the two agents. Our trial was conducted in adults rather than children. PNs grow most rapidly in children,^9^ and growth rate tends to decline by adulthood. In fact, only 10.5% of patients in our trial enrolled because of tumor progression. Since the PNs in younger patients may be more susceptible to tumor shrinkage from a targeted agent, the lower response rate in our trial may be secondary to patient selection. Unlike the selumetinib trial, which followed a formal phase I study to determine the maximal tolerated dose for children with NF1 and PN, no such formal determination of maximal tolerated dose for adults with NF1 and PN was performed before launching this trial. Thus, the dose chosen might have been the minimum effective dose, which is borne out by the lack of responders among those with dose reductions. In addition, our trial was designed to give a rapid readout of efficacy; therefore, patients who did not have 15% tumor shrinkage after eight courses, or 20% shrinkage by 12 courses, were removed from the study. The selumetinib PN trial did not contain these requirements, and patients on the recommended phase II dose had a median time to best response of 22 courses. A different trial design that allowed for patients to stay on study for up to 24 courses as long as they did not progress might have allowed for a higher response rate to mirdametinib.

The PK data suggest that the dose chosen seems to be at the minimum effective dose. Dosing above 2 mg/m^2^/dose might result in more responses, as we found a potential relationship between mirdametinib exposure and tumor response. Patients who received near the higher end of dosing seemed more likely to respond. One of the challenges in this study was the availability of only 1 mg capsules, which necessitated a range of dosing of 1.8-2.2 mg/m^2^/dose, depending on the size of the patient. The availability of different formulations (capsule sizes or liquid) would allow for more precise dosing. Finally, the PK data imply that although tumor response is associated with drug exposure, drug toxicity resulting in dose reductions is not; thus, a higher dose might be tolerable, perhaps allowing a higher drug exposure.

Mirdametinib was safe and tolerable at the doses used in this clinical trial. Dose reductions occurred in 26.3% of patients, but these were mostly due to nonsevere side effects like grade 1 rash. In addition, four patients withdrew from the study for intolerable low-grade rash. No patients experienced a DLT.

The PRO results should be considered exploratory since this study was not powered to draw conclusions about changes in these measures. However, significant and clinically meaningful decreases in tumor pain intensity occurred in the total group, which persisted through course 12 in the patients who experienced a PR similar to other MEKi trials.^3,4^ The PR group also exhibited significant and durable decreases in the interference of pain in daily life. In preclinical studies, mechanisms of neuropathic pain involve activation of the MEK/ERK pathway in neurons, microglia, and astrocytes,^15,16^ and MEK inhibition may decrease pain in part by reducing inflammatory pain hypersensitivity^17^ and microgliosis.^15^ Furthermore, the PR group reported some improvements in physical functions, but a worsening in skin problems, likely related to the acneiform rash. The improvements in the cognitive domain are intriguing as drugs targeting the Ras/MAPK pathway may positively affect cognitive function in NF1,^18^ and PD-0325901 crosses the blood brain barrier better than other MEKi drugs.^19^ Animal models suggest that the ERK/MAPK pathway plays a role in neuronal plasticity by modulating GABA release, long-term potentiation, and hippocampal-dependent learning and memory.^18,20^ The use of prospectively administered PRO measures was feasible in this multicenter trial and should be included in future studies.

Limitations of this trial include the small sample size, the single capsule dose available, lack of dose optimization before start of study, and the lack of functional testing performed. In addition, our trial design, which minimized the patients who remained on protocol therapy without a clear response, might have inadvertently missed late responders to mirdametinib, as evidenced by several responders who did not reach maximal response by course 12. As the study did not require off-treatment MRI scans, we were unable to assess the durability of response once mirdametinib was stopped. Future trials of mirdametinib should eliminate the requirement of early removal for not achieving 15% volume reduction by course 8, should consider allowing patients to remain on therapy beyond course 12 even if they have not yet achieved a PR, and should mandate tumor volume assessments following discontinuation of medication to assess for durability of response.

In conclusion, this trial demonstrated that mirdametinib is safe and effective in adolescent and adult patients with NF1-associated PNs. A larger trial further examining this agent in both children and adults with NF1 and PNs is currently underway (ClinicalTrials.gov identifier: NCT03962543). Future trials might consider optimizing the dosing of mirdametinib for tumor efficacy, testing MEK inhibition in combination with other agents to find therapies that will increase the response rate, and examining this agent's effect on cognitive functioning.

## Figures and Tables

**TABLE A1.**
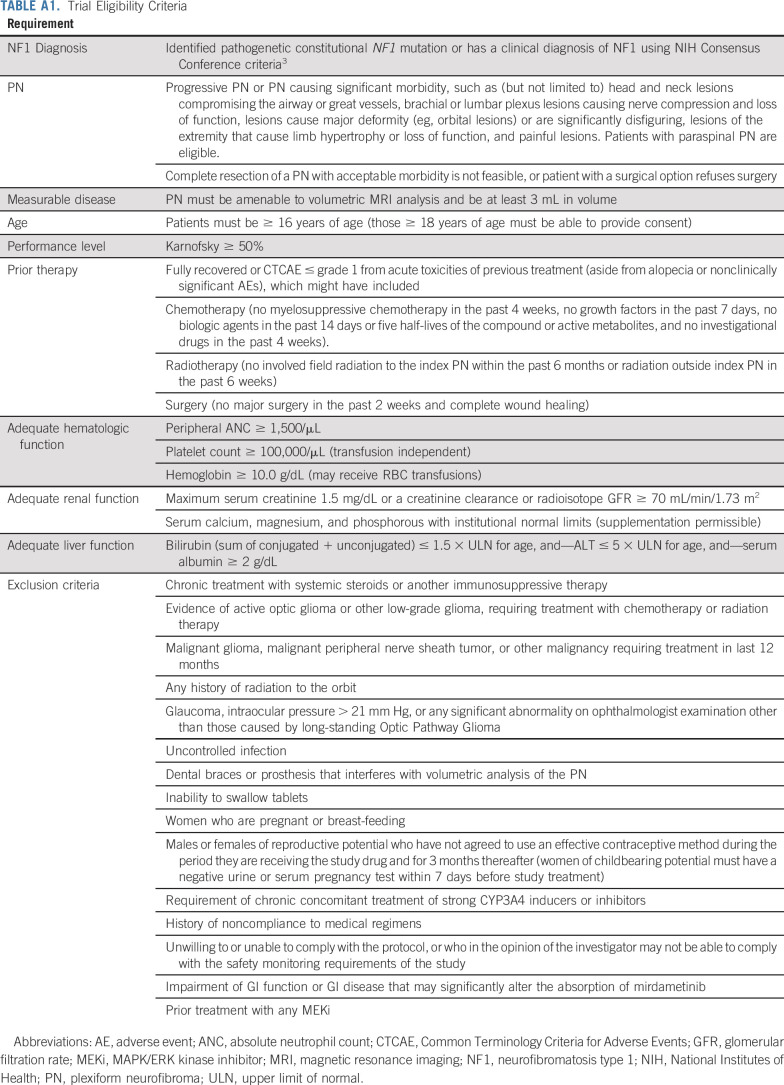
Trial Eligibility Criteria

**TABLE A2.**
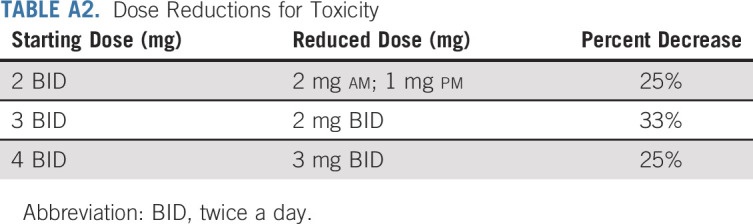
Dose Reductions for Toxicity

**TABLE A3.**
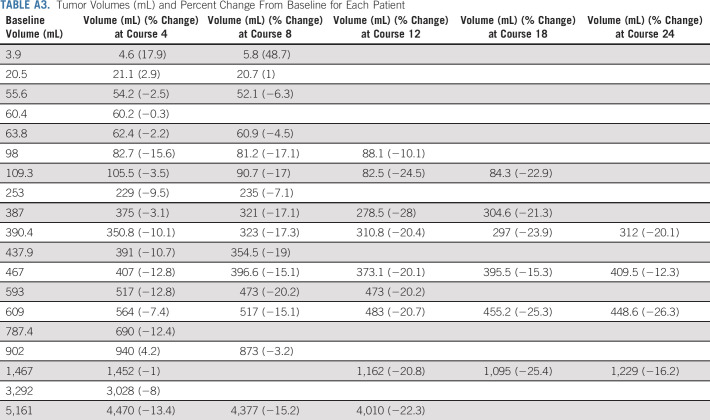
Tumor Volumes (mL) and Percent Change From Baseline for Each Patient

**TABLE A4.**
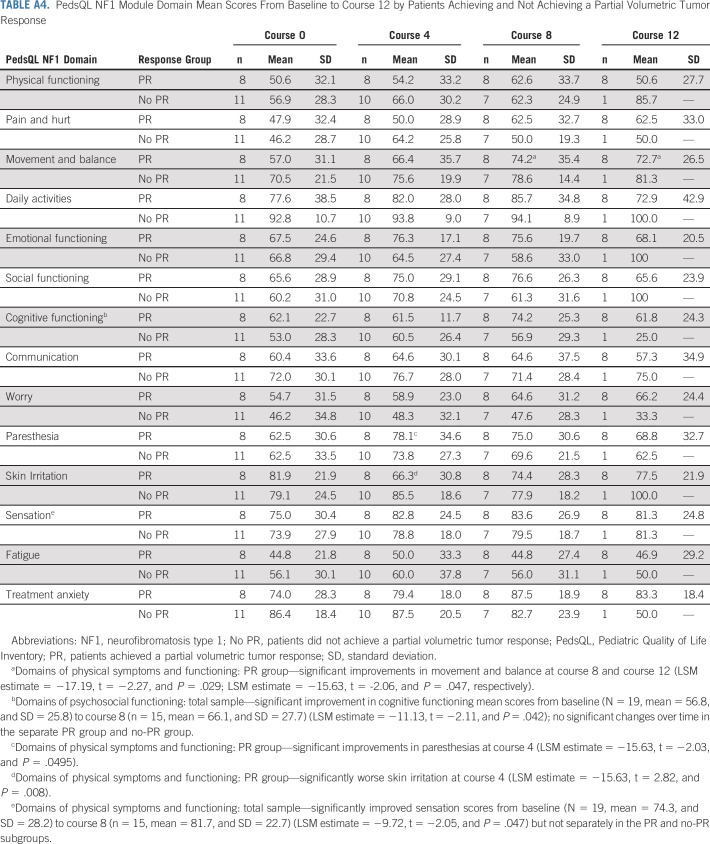
PedsQL NF1 Module Domain Mean Scores From Baseline to Course 12 by Patients Achieving and Not Achieving a Partial Volumetric Tumor Response
